# Second lesions located within the same belt-like region along the stomach's short axis as primary lesions: Boundary equal lesion trends

**DOI:** 10.1055/a-2789-1092

**Published:** 2026-02-05

**Authors:** Toshifumi Iida, Yoshiaki Kimoto, Etsuko Yamabe, Miuzen Kanamori, Susumu Banjoya, Tomoya Kimura, Koichi Furuta, Shinya Nagae, Hiroshi Yamazaki, Nao Takeuchi, Shunya Takayanagi, Takafumi Konishi, Yuki Kano, Kohei Ono, Ryoju Negishi, Yohei Minato, Hideyuki Chiba, Ken Ohata

**Affiliations:** 1 Department of Gastrointestinal Endoscopy, NTT Medical Center Tokyo, Shinagawa-ku, Japan; 2 215674 Gastroenterology, Itabashi Chuo Medical Center, Itabashi, Japan; 3 60498 Medicine, Haukeland University Hospital, Bergen, Norway; 4 36845 Department of Gastroenterology, Rinku General Medical Center, Izumisano, Japan; 5 215674 Itabashi Chuo Medical Center, Itabashi, Japan; 6 Gastroenterology, Omori Red Cross Hospital, Ota-Ku, Japan

**Keywords:** Endoscopy Upper GI Tract, Endoscopic resection (ESD, EMRc, ...), Malignant strictures, Diagnosis and imaging (inc chromoendoscopy, NBI, iSCAN, FICE, CLE), GI surgery

## Abstract

**Background and study aims:**

Gastric adenoma and cancer are common in Asia, with early detection critical for prognosis. Synchronous multiple early gastric cancers (SMEGCs) occur in 6% to 14% of cases, but their clinicopathological characteristics remain unclear. This study analyzed synchronous multiple gastric neoplasms treated by endoscopic resection or surgery to aid early detection.

**Patients and methods:**

Among 2,991 cases of early gastric cancer or adenoma diagnosed at our institution, 173 patients with 346 synchronous lesions (January 2016-March 2024) were analyzed. All lesions were mucosal or submucosal. Lesions were categorized as “1st” (larger) and “2nd” (smaller), and clinicopathological characteristics were compared using Chi-square and Fisher’s exact tests with Cramér’s V.

**Results:**

Patients had a mean age of 73.2 years; 72.8% were male. Most lesions were in the lower/middle stomach, differentiated (92.2%), depressed (52.9%), and brownish on narrow-band imaging (65.3%). Mean tumor diameter was 13.4 mm. Although 1st lesions were larger, other features showed high concordance (≥ 0.25 Cramér’s V) in location, morphology, histology, invasion depth, and coloration. Survival was 94.8% (nine unrelated deaths).

**Conclusions:**

Synchronous multiple gastric neoplasms tend to have similar endoscopic and histopathologic features and often occur within the same belt-like region along the short axis of the stomach. This pattern was named boundary equal lesions trends (BELT). When detecting one lesion, considering BELT is essential.

## Introduction

Gastric adenoma and early gastric cancer are commonly encountered neoplasms, not only in Japan but also in other Asian countries. In recent years, advancements in endoscopic diagnostic techniques and widespread implementation of gastric cancer screening have significantly enhanced detection rates for these diseases. In addition, it has often been reported that multiple neoplasms are often detected simultaneously, with such cases referred to as synchronous multiple early gastric cancers (SMEGCs) 1 .

The predominant risk factor for gastric cancer is primarily attributed to infection with *Helicobacter pylori* , which induces field cancerization, resulting in a strong tendency for synchronous cancer development. Early gastric cancer is associated with an exceptionally low risk of lymph node metastasis, and endoscopic submucosal dissection (ESD) has become the globally accepted standard treatment for gastric adenomas and intramucosal carcinomas 2 3 . Surgery is the preferred approach for submucosal invasive cancer. Altogether, for lesions classified as early gastric cancer, both ESD and surgery generally provide an excellent prognosis 4 .

According to previous reports, SMEGC occurs at a frequency of between 6% and 14% of all gastric cancers in Asia 5 6 7 . However, these estimates were derived from studies conducted approximately a decade ago, when prevalence of *H. pylori* infection was significantly different from the current trends. Furthermore, previous studies of SMEGC were often focused exclusively on cases treated by surgery or endoscopic techniques and are either outdated or limited by selection bias 1 8 9 .

For these reasons, previous reports may not accurately reflect current prevalence of *H. pylori* infection or include the entire spectrum of early gastric cancer because their cases were limited to endoscopic or surgical treatment. In this study, we retrospectively evaluated clinicopathologic characteristics of patients with gastric adenomas and carcinomas that were histopathologically confirmed as having synchronous multiple gastric neoplasms. The present study aimed to comprehensively investigate clinicopathological features of synchronous multiple gastric neoplasms by including cases treated with both endoscopic and surgical procedures, thereby providing a more accurate reflection of current clinical practice and *H. pylori* infection prevalence. Specifically, we hypothesize that synchronous multiple gastric neoplasms within the same patient share similar clinicopathological features and tend to occur within a specific, spatially related "belt-like" region of the stomach, reflecting the concept of field cancerization. This study aimed to objectively interpret our data to support or refute this hypothesis.

## Patients and methods

### Study design

This study was designed as a retrospective cohort study to investigate clinicopathological characteristics of synchronous multiple gastric neoplasms. All procedures were performed at the NTT Medical Center Tokyo. The study was conducted with the approval of the Institutional Review Board of NTT Medical Center Tokyo. (IRB No. 000200015936–01). All authors had access to the study data and reviewed and approved the final manuscript.

### Patients

Among 2,991 consecutive patients whose information was collected from a prospectively managed database and who had been diagnosed as having early gastric cancer or adenoma between January 2016 and April 2024, we retrospectively analyzed data from 173 patients (346 lesions) who met the following criteria. Inclusion criteria were: 1) patients diagnosed as having two synchronous early gastric cancers or adenomas based on the pathological findings in resected specimens; and 2) patients confirmed as having tumors confined to the mucosal or submucosal layers in the pathology specimens after resection. Exclusion criteria were: 1) patients who had received preoperative chemotherapy; 2) patients with insufficient pathologic or clinical information; 3) patients with three or more synchronous multiple gastric neoplasms; and 4) patients in whom a second lesion was detected more than 1 month after the initial lesion and within 1 year.

The rationale for these exclusion criteria is as follows: Patients with advanced gastric cancer were excluded to focus specifically on early-stage synchronous multiple gastric neoplasms where curative endoscopic or surgical treatments are predominantly applied and prognosis is generally excellent. Lesions numbering more than two were excluded in this initial phase of the study to simplify analysis of spatial distribution and clinicopathological concordance between pairs of synchronous lesions, allowing for a focused investigation of the boundary equal lesion trends (BELT) hypothesis without confounding factors of complex multifocal presentations. Furthermore, lesions detected between 2 to 12 months after the initial lesion, although sometimes considered metachronous, were excluded to ensure a strict definition of synchronicity within a narrow timeframe (detection within 1 month), minimizing the possibility of truly metachronous lesions being misclassified as synchronous due to delayed detection rather than true synchronous development. This strict definition enhanced the homogeneity of the study cohort and strengthened the assessment of synchronous lesion characteristics. However, we acknowledge that excluding cases with more than two lesions may limit generalizability to more extensive field cancerization scenarios, which will be addressed in the Discussion section as a study limitation and an area for future research.

**Fig. 1:**
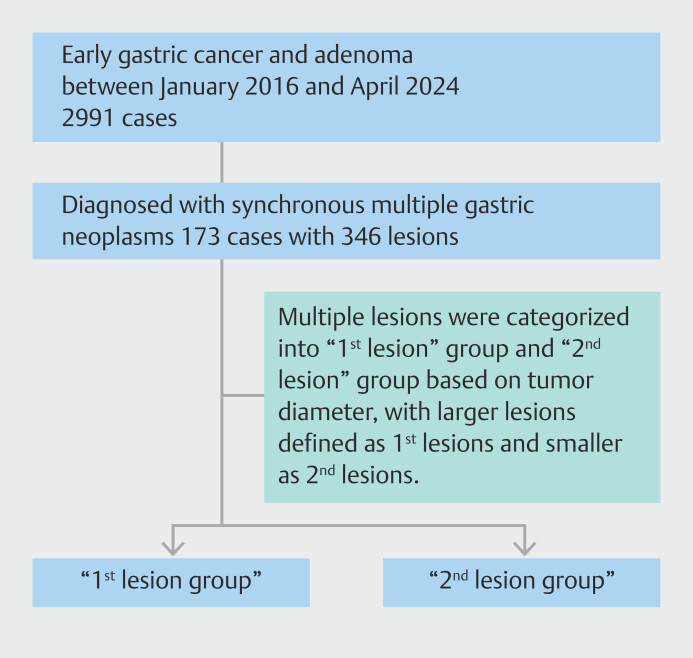
Flowchart of patient enrollment.

Multiple lesions were categorized into 1st lesion and 2nd lesion groups based on the tumor diameter, with larger lesions defined as 1st lesions and smaller ones as 2nd lesions ( Fig. 1 ). It is important to clarify that 1st lesion and 2nd lesion refer solely to their relative size in each synchronous pair, not to the chronological order of their occurrence or detection. This nomenclature was adopted to facilitate comparison of clinicopathological features between a larger and a smaller lesion within the same patient.

### Definitions of clinicopathological characteristics

Lesion morphology was defined according to the Paris classification, based on endoscopic findings (elevated, flat, depressed) 10 . Lesion location was described as in the lower third, middle third, or upper third of the stomach according to the Japanese Classification of Gastric Carcinoma 11 . The middle third and lower third of the stomach were each equally divided into two sections—anal side (1) and oral side (2)—resulting in a total of five anatomical regions. Lesion size was measured during pathological examination (maximum diameter). Lesion coloration was defined as reddish, same, or discolored, compared with surrounding mucosa. Severity of atrophic gastritis was classified as mild (C-0 to C-2), moderate (C-3 to O-1), or severe (O-2 to O-3) according to the Kimura and Takemoto classification 12 13 14 15 . It should be noted that the Kimura-Takemoto classification typically starts from C-1 for mild atrophy. In this study, C-0 was used to denote cases with no apparent atrophy or minimal changes not fitting into C-1 or higher, representing the mildest end of the spectrum. Histological assessments were made by pathologists who were board-certified by the Japanese Society of Pathology. For the purpose of clinicopathological correlation within this study, lesions were further categorized as differentiated or undifferentiated types based on the World Health Organization classification of gastric adenocarcinoma, which is commonly used in pathological practice to describe tumor histology alongside the Vienna Classification (VCL) 16 . High-grade dysplasia in this context refers to VCL categories 4.2 and 4.3, consistent with severe dysplasia or carcinoma in situ, as per common pathological usage in Japan, which often considers these entities distinct from invasive carcinoma.

### Statistical analysis

All continuous variables are expressed as means ± standard deviation (SD) or median values and compared by Student’s *t* -test or Mann-Whitney’s U test. All categorical variables are described as frequencies (percentage) and the chi-square test or Fisher’s exact test was used to compare them. In addition, the strength of the associations with the clinicopathologic features was calculated. For this purpose, Cramer’s V coefficient (Vc) values were calculated. Vc was used to evaluate the strength of association. The degree of association was considered to indicate a weak association for values between 0.10 and 0.25, a moderate association for values between 0.25 and 0.50, and a strong association for values between 0.50 and 1.00. These thresholds for Cramer's V were adopted based on established guidelines for interpreting effect sizes in categorical data analysis. SPSS v25.0 was used for the statistical analysis (IBM Corp. IBM SPSS Statistics for Windows, Version 25, New York, United States). The level of significance was set at *P* < 0.05. Normality of continuous variables was assessed using the Shapiro-Wilk test. Variables not following a normal distribution were presented as median (interquartile range) and compared using the Mann-Whitney U test, whereas normally distributed variables were expressed as mean ± SD and compared using the Student's *t* -test.

## Results

Data from a total of 173 patients with 346 lesions were included in this analysis. Clinicopathological characteristics of the patients/lesions are shown in Table 1 . The patient population was predominantly male (72.8%, 126/173), with a mean age of 73.2 ± 9.7 years. *H. pylori* infection status was “current infection” in 28.9% (50/173), “past infection” in 56.1% (97/173), and “never infection” in 15.1% (26/173) of patients. Severity of the underlying gastric mucosal atrophy was classified as mild in 9.2% (16/173), moderate in 37.0% (64/173), and severe in 53.8% (93/173) of patients. These observations imply that the majority of patients had a background of atrophic gastritis related to *H. pylori* infection. Hypertension was the most common comorbidity (37.6%).

**Table:** **Table 1** Baseline clinical characteristics of patients.

	n	SD or %
Age, years, mean (SD)	73.2	9.7
Gender, male, n (%)	126	72.8%
Presence of *Helicobacter pylori* infection, n (%)		
Current	50	28.9%
Past	97	56.1%
Never	26	15.0%
Past medical history, n (%)		
Hypertension	65	37.6%
Diabetes mellitus	24	13.9%
Cardiac diseases	22	12.7%
Cerebral diseases	14	8.1%
Liver cirrhosis	1	0.6%
Chronic kidney disease	3	1.7%
Smoking, n (%)	49	28.3%
Alcohol, n (%)	51	29.5%
Medication		
Antithrombotic drug, n (%)	42	24.3%
PPI, n (%)	53	30.6%
Endoscopic findings		
Map like redness, n (%)	81	46.8%
Atrophic gastritis according to the endoscopic examinations, n (%)		
Mild (C-0~C-2)	16	9.2%
Moderate (C-3~O-1)	64	37.0%
Severe (O-2~O-3)	93	53.8%
Postoperative stomach, n (%)	2	1.2%
PPI, proton pump inhibitor; SD, standard deviation.		

Medications that patients were taking included antithrombotic agents (24.3%, 42/173) and proton pump inhibitors (30.6%, 53/173). Map-like redness was observed in 46.8% of patients (81/173) and postoperative stomach was noted in 1.2% of patients (2/173) ( Table 1 ). During the observation period, the survival rate was 94.8%, with nine deaths, all due to unrelated causes. The observation period for survival analysis extended from the date of initial diagnosis of synchronous multiple gastric neoplasms to the date of last follow-up or death, with median follow-up of 5.35 years (1.11–9.20 years).

### Characteristics of synchronous multiple gastric neoplasms

A total of 346 lesions were analyzed. Characteristics of the synchronous multiple gastric neoplasms are shown in Table 2 . Mean tumor diameter was 13.4 ± 10.1 mm. Distribution of tumor locations was as follows: lower third 1 (L1) 25.4% (n = 88), lower third 2 (L2) 22.0% (n = 76), middle third 1 (M1) 24.6% (n = 85), middle third 2 (M2) 17.9% (n = 62), and upper third (U) 10.1% (n = 35). In terms of surface orientation, tumors were located along the lesser curvature in 41.3% (n = 143), along the anterior wall in 19.1% (n = 66), along the greater curvature in 23.4% (n = 81), and along the posterior wall in 16.2% (n = 56) of patients. Histopathologic findings included HGD in 4.0% (n = 14), differentiated carcinoma in 92.2% (n = 319), and undifferentiated carcinoma in 3.8% (n = 13). Ulceration (UL) was observed in 1.4% of lesions (n = 5). Regarding depth of invasion, the tumor was confined to the mucosa in 87.0% (n = 301), submucosal invasion depth was < 500 µm in 4.6% (n = 16), and submucosal invasion depth was ≥ 500 µm in 4.3% of patients (n = 15) and HGD was noted in 4.0% of patients (n = 14). Morphologically, lesions were elevated in 42.8% (n = 148), flat in 4.3% (n = 15), and depressed in 52.9% of patients (n = 183). Endoscopic examination revealed a brownish area (BA) on narrow-band imaging (NBI) in 65.3% of patients (n = 226). In regard to lesion color, lesions were reddish in 54.3% (n = 188), the same color as the surrounding mucosa in 15.9% (n = 55), and showed discoloration in 29.8% of patients (n = 103) ( Table 2 ).

**Table:** **Table 2** Clinicopathologic comparison between the “1st Lesion” Group and “2nd Lesion” Group.

	Total		1st Lesion		2nd Lesion		*P* value
	n	SD or %	n	SD or %	n	SD or %	
Size, mm, mean (SD)	13.4	10.1	17.5	10.62	9.2	7.56	< 0.05
Locations, n (%)							
Lower third 1	88	25.4%	48	27.7%	40	23.1%	0.340
Lower third 2	76	22.0%	33	19.1%	43	24.9%	
Middle third 1	85	24.6%	40	23.1%	45	26.0%	
Middle third 2	62	17.9%	34	19.7%	28	16.2%	
Upper third	35	10.1%	18	10.4%	17	9.8%	
les	143	41.3%	72	41.6%	71	41.0%	0.545
ant	66	19.1%	29	16.8%	37	21.4%	
gre	81	23.4%	40	23.1%	41	23.7%	
pos	56	16.2%	32	18.5%	24	13.9%	
Pathologic results of lesions, n (%)							0.246
High-grade dysplasia	14	4.0%	4	2.3%	10	5.8%	
Differentiated carcinoma	319	92.2%	163	94.2%	156	90.2%	
Undifferentiated carcinoma	13	3.8%	6	3.5%	7	4.0%	
UL, n (%)	5	1.4%	4	2.3%	1	0.6%	0.186
Depth of invasion, n (%)							0.083
High grade dysplasia	14	4.0%	4	2.3%	10	5.8%	
Mucosa	301	87.0%	149	86.1%	152	87.9%	
Submucosal invasion depth < 500 µm	16	4.6%	12	6.9%	4	2.3%	
Submucosal invasion depth ≥ 500 µm	15	4.3%	8	4.6%	7	4.0%	
Morphology, n (%)							0.375
Elevated	148	42.8%	73	42.2%	75	43.4%	
Flat	15	4.3%	5	2.9%	10	5.8%	
Depressed	183	52.9%	95	54.9%	88	50.9%	
Endoscopic findings							
Brownish area (NBI), n (%)	226	65.3%	113	65.3%	113	65.3%	1
Color							0.643
reddish, n (%)	188	54.3%	98	56.6%	90	52.0%	
same, n (%)	55	15.9%	25	14.5%	30	17.3%	
discolor, n (%)	103	29.8%	50	28.9%	53	30.6%	
UL; ulceration.							

### Comparison of 1st and 2nd lesion groups

A total of 173 cases in each of the 1st and 2nd lesion groups were analyzed. Mean tumor diameter was larger in the 1st lesion group as compared with the 2nd lesion group (17.5 mm vs. 9.2 mm, *P* < 0.05). This observation is consistent with the definition of 1st lesion as the larger lesion in each pair, as described in the patients and methods section. Tumor locations were similar between the two groups, with the majority located in the lower and middle thirds of the stomach. Histopathologically, the percentage of differentiated carcinomas was the highest in both lesion groups, whereas there was a slightly higher number of lesions showing HGD in the 2nd lesion group as compared with the 1st lesion group. Both groups had comparable distributions of morphologic features, with elevated and depressed lesions being the most common in both groups. Endoscopic findings, including presence of BA and lesion coloration, were also similarly distributed between the two groups ( Table 2 ).

Cramer's V was 0.632 for location (L1/2/M1/2/U), 0.481 for location (lagp), 0.220 for the histopathology, 0.438 for morphology, 0.404 for depth of invasion, 0.796 for BA, and 0.768 for coloration. These results revealed associations between the two groups for all parameters examined. Furthermore, the Cramer's V value exceeded 0.25 for six items (location (L1/2/M1/2/U), location (lagp), morphology, depth of invasion, BA, coloration), indicating particularly strong associations ( Table 3 ). In our cohort of 173 patients, the proportion of cases where both the 1st and 2nd lesions were located within the same BELT-defined region (e.g., lower third, middle third, or upper third) was approximately 80% (138/173 cases).

**Table:** **Table 3** Concordance rates and Cramer’s V values of clinicopathological findings.

	Concordance rates, %(n)	X2 (p)	Vc
Location (L1/2/M1/2/U)	77.5 (134)	276.22 (< 0.01)	0.632
Location (L/A/G/P)	63.0 (109)	120.18 (< 0.01)	0.481
Histology	87.9 (152)	16.69 (0.002)	0.220
Morphology	74.6 (129)	66.48 (< 0.01)	0.438
Depth of invasion	82.7 (143)	84.69 (< 0.01)	0.404
BA	90.8 (157)	109.58 (< 0.01)	0.796
Coloration	86.1 (149)	204.02 (< 0.01)	0.768
BA; brownish area; Vc, Cramer’s V.			

## Discussion

Gastric cancer is the most commonly diagnosed cancer worldwide, with the highest incidences reported from East Asia and Eastern Europe. It is the fifth most common cancer in the world in terms of incidence and the fourth most common in terms of mortality 17 . On the other hand, treatments for gastric cancer have also advanced significantly. In particular, endoscopic therapy for early-stage gastric cancer has come to be widely adopted, especially in Asia. Earlier T categories (in the TNM classification) are generally associated with better long-term outcomes after treatment 18 . For this reason, identifying gastric cancer at an early stage or even at a precancerous stage is of crucial importance to provide good patient prognosis. Moreover, gastric cancer is often linked to *H. pylori* infection and frequently shows a tendency toward multifocal development. When one lesion is detected, it is important to carefully check for presence of additional lesions to ensure thorough evaluation and management. Several reports have estimated that frequency of synchronous multiple gastric cancers treated by endoscopy ranges from 2.5% to 14.0% 19 20 21 . In a study of early and advanced synchronous multiple gastric cancers treated by radical gastrectomy, frequency of synchronous multiple gastric cancers was reported as 4.8% to 23.8% 22 23 . However, these reports were based on only cases treated by surgical resection and entailed selection bias in relation to treatment method. Moreover, these reports were also based on older data, making them less reflective of the current prevalence of *H. pylori* infection. Therefore, we conducted an analysis of incidence of SMEGCs encountered at our institution over a defined period. In our database, frequency of synchronous multiple gastric neoplasms treated with endoscopy or surgery was 6%, which is consistent with previous reports. The synchronous multiple gastric neoplasms in this study were associated with current or past *H. pylori* infection in 80% of cases and 90% of cases had moderate or more severe gastric atrophy. In most cases, the tumors were differentiated carcinomas, with only a few rare cases of undifferentiated carcinoma. Approximately half the lesions showed map-like redness, and the 2nd lesions were less than 10 mm in diameter, indicating that identification of synchronous multiple gastric neoplasms is often challenging and these lesions easily can be missed. Many previous reports have highlighted risk factors for synchronous multiple gastric neoplasms and have warned of missed cancers. Specific risk factors include male gender, undifferentiated type of carcinoma, and tumors located in the lower third of the stomach, which could also be considered as predictive factors for SMEGC in patients undergoing endoscopic resection 1 23 24 25 .

Previous reports have often compared patients with and without SMEGC or compared overall trends between initially detected lesions and missed lesions 25 26 27 28 29 . However, few reports have discussed similarity of the sites of occurrence and endoscopic findings of synchronous multiple gastric neoplasms within the same patient. As for recent studies examining the site of occurrence, Hayashi et al. published a paper in 2024 on the positional relationship between primary and secondary lesions of SMEGC 30 . However, in the study reported herein, only cases treated by ESD were included, which could have led to a bias toward mucosal cancers among cases of early gastric cancer. Also, cases resected by EMR or polypectomy, as well as those which required surgical resection due to technical difficulties such as ulcer scars, may have been excluded. Therefore, because the selection criteria could have introduced a significant selection bias, their findings may not accurately reflect the pure biological characteristics of SMEGCs. Although Hayashi et al.'s study provides valuable insights into the spatial relationships of SMEGCs, its focus on only ESD cases might introduce a selection bias toward mucosal lesions, potentially limiting generalizability to the broader spectrum of early gastric cancers 30 . In contrast, our study included not only endoscopically treated cases, but also surgically treated cases so that we could target all cases of early gastric cancers, and we were able to include 173 patients with a total of 346 lesions.

In this study, synchronous multiple gastric neoplasms were often reddish or showed discoloration as compared with surrounding mucosa. When classified by the Unified Medical Language region of origin, the neoplasms occurred in the same region in about 80% of cases. Concordance rates for the pathological findings, tumor morphology, depth of invasion, BA, and lesion color were also high, suggesting that synchronous multiple gastric tumors show similar characteristics. Cramer’s V values were calculated for each of the items using the chi-square test; details are shown in Table 3. Significant associations were noted for all the items (Cramer’s V > 0), indicating the existence of correlations between the two groups and suggesting that synchronous multiple gastric tumors show similar findings and characteristics. In particular, Cramer’s V values exceeded 0.25 for location (UML), location (lagp), morphology, depth of invasion, BA, and coloration, indicating high degrees of correlation. Although a Cramer's V value of 0.25 indicates a moderate association according to Cohen's guidelines, the consistent observation of V values exceeding this threshold across multiple crucial clinicopathological features, coupled with values approaching 0.8 for BA and coloration, collectively points to a clinically significant pattern of concordance. This suggests that synchronous multiple gastric tumors arise in close proximity within the same mucosal environment and share similar clinicopathological features, supporting the field cancerization theory. Notably, it is well established that background mucosal inflammation induced by *H. pylori* infection is a risk factor for gastric carcinogenesis. In *H. pylori* -infected stomachs, gastric mucosal atrophy is thought to progress sequentially from the pyloric side to the lesser curvature of the gastric body and then to the greater curvature, which led Kimura and Takemoto to classify atrophy severity 15 . Based on this, it would be reasonable to expect that the degree of inflammation in the background mucosa, which serves as the cancer precursor, is similar in regions in close proximity to each other. Indeed, in this study, gastric tumors occurred within the atrophic mucosa in 95.1% of cases (329/346).

**Fig. 2:**
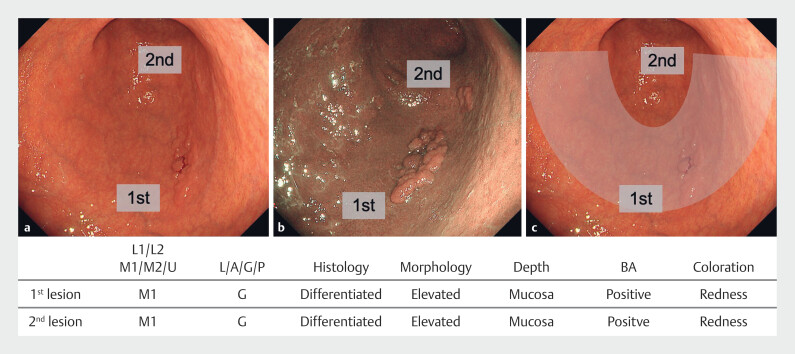
A case of “BELT: Boundary Equal Lesion Trends”. An 89-year-old, female patient. **a** White light imaging. **b** Narrow band imaging. **c** White light imaging with the “BELT” line.

This study showed that synchronous multiple gastric neoplasms have similar endoscopic and histopathologic features and often tend to occur within the same belt-like region along the short axis of the stomach. The tumors that occur in multiple lesions in this area, therefore, were given the name BELT. This strong spatial concordance supports the BELT hypothesis. An example of a typical case is shown in Fig. 2 . Findings in this case were consistent with histopathological and endoscopic findings reviewed in our study. In patients diagnosed as having gastric cancer by screening upper gastrointestinal endoscopy, it is important to be aware of BELT to detect any tumors with similar features and gross findings. This study included both cases treated by endoscopic and surgical procedures and compared multiple lesions with each other. In other words, the study is of important clinical significance because it included all neoplasms up to submucosal invasive gastric cancer (T1). The sample size was also adequate, because in our opinion, this sample was sufficient for a study of relevance. Limitations of this study were that it was conducted in patients from a single institution and had a retrospective design, which could have introduced information bias. Also, patients with three or more synchronous lesions were initially excluded due to concerns about increased complexity and potential confounding. Specifically, such cases may include a mixture of truly synchronous and potentially missed lesions, making it difficult to accurately assess the spatial distribution and synchronicity of tumors. However, we acknowledge that inclusion of these cases could provide additional insights into the spatial clustering of lesions and support the BELT hypothesis. Future studies will be needed to evaluate this subset more comprehensively.

Another limitation stems from the inherent challenge of precisely defining synchronous lesions versus metachronous lesions in clinical practice. Although our study employed a strict 1-month interval criterion to define synchronicity, it is possible that some lesions evolving rapidly within this timeframe or missed during an earlier endoscopy could theoretically confound analysis of true simultaneous development. Future research with serial high-definition endoscopic surveillance or advanced imaging techniques could further refine this distinction.

## Conclusions

In conclusion, synchronous multiple gastric tumors have similar endoscopic and histopathologic features and tend to occur in the same region of the stomach. In patients presenting with a gastric tumor, it is important to consider “BELT” to detect any synchronous lesions. This concept has significant clinical implications. Endoscopists should be particularly vigilant in thoroughly inspecting the "BELT" region—the same short-axis belt where a primary lesion is found—for additional subtle lesions, even after detecting an initial gastric neoplasm. This enhanced awareness of spatial clustering and shared characteristics could lead to improved detection rates for synchronous lesions, thereby optimizing patient management and prognosis. For instance, if an initial lesion is found in the lower third along the lesser curvature, careful observation of this entire "belt" (around the circumference in the lower third) should be performed using advanced endoscopic techniques such as NBI, specifically looking for reddish or discolored lesions with similar morphology.
